# Screening of Monokaryotic Strains of *Ganoderma sichuanense* for Gene Editing Using CRISPR/Cas9

**DOI:** 10.3390/jof12010025

**Published:** 2025-12-28

**Authors:** Le Li, Yuxuan Liu, Jianzhong Wu, Nuan Wen, Yang Song, Xue Wang, Zhuang Li, Huiying Sun, Yongping Fu

**Affiliations:** 1 College of Mycology, Jilin Agricultural University, Changchun 130118, China; 2 College of Agriculture, Jilin Agricultural University, Changchun 130118, China; 3 College of Plant Protection, Shandong Agricultural University, Tai’an 271018, China

**Keywords:** *Ganoderma sichuanense*, monokaryotic strain, CRISPR/Cas9, gene editing

## Abstract

*Ganoderma sichuanense* is a widely used medicinal and edible fungus. Genomic studies have revealed substantial genetic variation among its different strains, indicating that a genetic transformation system optimized for one genotype may not be effective in others. However, no study has systematically evaluated the efficiency of a genetic transformation system across diverse genotypes, which has potentially limited functional genetic studies in this species. In this study, we first evaluated eight wild and cultivated monokaryotic strains with different genotypes based on their hygromycin B resistance and green fluorescent protein (GFP) expression efficiency. Three strains (CCMJ1500101, CCMJ1509001, and CCMJ1507802) were identified as capable of stable foreign gene expression, achieving transformation efficiencies of 20.0–66.7% via PEG-mediated protoplast transformation. Subsequently, a CRISPR/Cas9 system incorporating seven key elements to enhance editing efficiency was constructed and applied to these three strains using the *ura3* gene as a test target. Gene editing efficiencies varied significantly among genotypes, ranging from 14.3% to 75.0%, confirming the system’s high efficacy and genotype dependence. Importantly, to rigorously assess the robustness and versatility of the established transformation platform, we further validated its broad applicability in the best-performing strain, CCMJ1500101, by successfully editing five functional genes involved in growth, development, and metabolism. Notably, gene inversion events were detected for the first time in edited transformants of *Ganoderma*, providing new clues for understanding non-homologous end joining (NHEJ) repair in this species. This study establishes a robust dual-sgRNA CRISPR/Cas9 platform for *G. sichuanense* and provides valuable strain resources to facilitate future gene functional studies and genetic improvement.

## 1. Introduction

*Ganoderma sichuanense* (syn. *G. lucidum* sensu auct. sina; *G. lingzhi*) is a macro-fungus belonging to Polyporales, Basidiomycota. Despite its edible values and diverse medicinal activities, analysis of gene functions and genetic improvement research of *G. sihuanense* has experienced considerable delays [1,2]. These delays are largely due to challenges such as heterokaryosis and poor compatibility with the existing gene-editing systems. In 2014, RNA interference (RNAi) was first applied to downregulate the expression of nicotinamide adenine dinucleotide phosphate oxidases (NOX) in *G. lucidum*, which verified their role in regulating ganoderic acid biosynthesis and hyphal branching [3]. Subsequently, this technology has served as the primary method for studying gene function in *Ganoderma* species, such as validating the regulatory roles of *GlPacC*, *GlSwi6B*, *GlPP2C1*, and *Glsirt1* genes in biological processes including mycelial growth, cell integrity, polysaccharide synthesis, and ganoderic acid biosynthesis [3,4,5,6]. However, RNAi generally results only in partial downregulation rather than complete knockout of the target gene, which in some cases may lead to an incomplete characterization of gene functions [7].

CRISPR/Cas9 is a novel targeted gene-editing technology that has been applied to numerous species in recent years, offering advantages such as simple operation, high precision, and low cost [8]. An effective gene-editing system requires efficient and compatible hosts, optimized vectors, enhanced promoters, and improved sgRNA stability, all of which are essential for achieving high editing efficiency. In 2017, the CRISPR/Cas9 system was first successfully implemented in *G. lucidum* by introducing the promoter of the endogenous glyceraldehyde-3-phosphate dehydrogenase (*gpd*) gene into the expression vector that significantly enhanced the expression level of target genes [9]. Subsequent studies have found that species-specific codon usage is critical for gene editing effectiveness, demonstrating that codon optimization along with a nuclear localization signal (NLS) can improve editing efficiency in *G. lucidum* [10]. Furthermore, introducing the *gpd* intron into the upstream of the *Cas9* gene was found to increase the frequency of CRISPR/Cas9-mediated gene disruption in *G. lucidum* [11]. Together, these studies underscore the critical role of vector design, especially by incorporating key genetic elements, in enhancing the efficiency of gene-editing systems.

It is also known that sustained expression of *Cas9* in vivo can increase genome editing efficiency. Meanwhile, using two sgRNAs (dual-sgRNA) rather than a single guide RNA (sgRNA) designed from those sequences flanking its target, on the other hand, is able to increase the feasibility of multiplex editing via the fragment deletion by dual-sgRNA-guided in vitro transcription [11]. The in vivo transcription of sgRNA ensures that *Cas9* has more sufficient sgRNA molecules for effective recognition and binding to target sequences than that of the in vitro transcription, thereby increasing the gene editing efficiency [12]. In fact, a CRISPR/Cas9 system capable of expressing sgRNA in vivo has already been developed, which facilitated the precise editing of a functional gene of *CYP5150L8* in *G. lucidum* via the homologous recombination (HR) [13]. Therefore, based on the above studies, employment of the dual-sgRNA in vivo transcription for targeted gene deletions could be a promising strategy for knocking out the gene function, especially when a low frequency of homologous recombination is considered.

So far, the CRISPR/Cas9 system has been applied to the functional analysis of six genes (*ura3*, *cyp5150l8*, *cyp505d13*, *GL17624*, *Ku70*, *pyrG*) involved in the biosynthesis pathways of pyrimidine nucleotides and ganoderic acids in *G. lucidum* [9,11,13,14,15,16,17]. Transcriptomic analysis of *G. lucidum* has predicted that 78 CYP450 genes are positively correlated with ganoderic acid synthesis [18]. Research on these CYP450 genes facilitates the understanding of the biosynthetic pathway of ganoderic acids [18]. The formation of fruiting bodies is a critical stage in the cultivation of edible fungi. From a genetic perspective, the mating type serves as the key factor that regulates the development of fruiting bodies in edible fungi, marking their entry into the sexual reproduction phase. Genetic manipulation in *G. lucidum* has long been a challenge, primarily because each connected hyphal cell contains two nuclei with distinct genetic components, and each nucleus differentially regulates cellular functions [17,19]. Monokaryotic strains of *G. lucidum*, which possess a single nucleus of uniform genetic origin, could be obtained through procedures such as protoplast preparation, protoplast regeneration, and monokaryon screening [17]. These monokaryotic strains are considered more suitable as efficient recipients for gene editing, since dikaryotic strains may reduce the editing efficiency due to partial genomic modifications [10]. Recipient strains reported so far in *G. lucidum* gene editing studies include the dikaryotic strain CGMCC 5.616 and its monokaryotic derivative 260125, the wild Korean strain GL3315 and one of its two monokaryons, and the monokaryotic strain L1 derived from the cultivated strain ‘Hunong No. 1’. Significant variations in editing efficiency have been reported when using different gene editing systems on these strains [9,11,13,15,16,17,20,21,22]. However, to date, no systematic screening of monokaryotic recipient strains based on the genotypes has been conducted in *G. lucidum* to identify those most effective for gene editing. This is particularly the case for approaches employing the dual-sgRNA in vivo genome-editing system carrying an expression vector constructed by integrating seven key cassettes for targeted gene deletions.

In this study, eight monokaryotic strains with different genotypes were evaluated based on their hygromycin resistance ability and *gfp* gene expression efficiency. Three monokaryotic strains, CCMJ1500101, CCMJ1509001, and CCMJ1507802, were identified as capable of foreign gene expression. Subsequently, using *ura3* as a model target, we applied an in vivo Cas9 expression and dual-sgRNA transcription strategy in these three monokaryotic strains to assess the feasibility and editing efficiency of the CRISPR/Cas9 system in *G. sichuanense*. Finally, five genes with various functions related to the growth, development, and metabolism of *G. sichuanense* were successfully edited in strain CCMJ1500101. This study not only established a functional dual-sgRNA in vivo transcription CRISPR/Cas9 gene editing system in *G. sichuanense*, but also revealed variations in genome editing efficiency among different monokaryotic strains. Strain CCMJ1500101, which showed comparatively high editing efficiency, demonstrates strong potential as an effective recipient for future effective gene editing studies. Therefore, this work provides a robust platform to accelerate functional gene analysis in this medicinal fungus.

## 2. Materials and Methods

### 2.1. Strains and Cultures

A total of eight monokaryotic strains of *G. sichuanense*, namely CCMJ1500101, CCMJ1503201, CCMJ15006301, CCMJ1506901, CCMJ1507201, CCMJ1507802, CCMJ1509001, and CCMJ1509302, were selected in the present study. Among them, CCMJ1500101, CCMJ1509001, and CCMJ1509302 were obtained from wild strains by monokaryotization through the isolation of protoplasts. All strains were preserved at Jilin Agricultural University. The monokaryotic strains of *G. sichuanense* were cultured in a malt yeast glucose (MYG) medium (ten g/L maltose, five g/L yeast extract, and five g/L glucose) at 28 °C in the dark. *Escherichia coli* DH5α (Shanghai Weidi Biotechnology Co., Ltd., Shanghai, China) was used for the plasmid construction and amplification.

### 2.2. Screening of Hygromycin Inhibitory Concentrations

To investigate the lethal concentration of hygromycin B, the mycelia of the above *G. sichuanense* strains were inoculated on the MYG medium containing 0, 25, 50, 100, 150, and 200 mg/L hygromycin B (Yeasen Biotechnology (Shanghai) Co., Ltd., Shanghai, China) for cultivation at 25 °C under dark conditions.

### 2.3. Construction and Activity Detection of Ganoderma Expression Vector

The *gfp* (green fluorescent protein) gene was selected as the target gene for the construction of a well-adapted expression vector in *G. sichuanense*. A constitutive promoter, Pgpd, was used to drive the expression of the *gfp* gene [9]. An intron, as reported previously, was introduced into the upstream region of the *gfp* gene to enhance its expression efficiency [11]. The GFP expression vector was designated as P203521. Dual verification was performed on the transformed strains as follows: (1) primary confirmation of transformants was carried out through screening on the antibiotic-resistant media, (2) detection of green fluorescent signals from the GFP protein under a fluorescence microscope (Hangzhou Chroma Optronics Co., Ltd., Hangzhou, China) that served as a standard for the expression evidence. These two processes ensured both the feasibility of genetic transformation in the strains and the ability to express exogenous genes. In this study, the well-adapted expression vector was introduced individually into eight monokaryotic strains of *G. sichuanense* for culture at the different hygromycin concentrations to screen the anti-hygromycin ability under the conditions mentioned above. Mycelia of individual strains grown in the MYG medium for five days were uniformly collected and used as experimental samples for the detection of *gfp* expression, which was conducted by using a fluorescence microscope with a 450–490 nm excitation filter, a 510 nm dichroic filter, and a 515 nm emission filter.

### 2.4. Construction of CRISPR/Cas9 Gene Editing Vector

**Figure 1:**
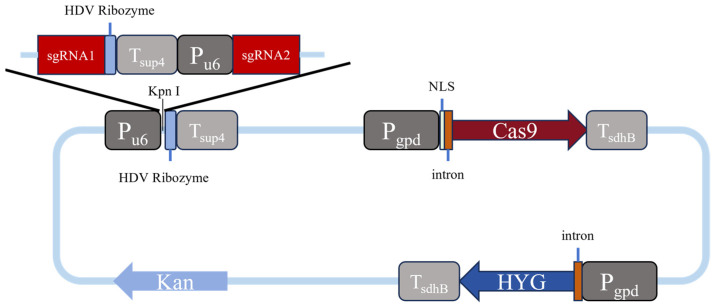
Schematic diagram of a gene editing vector.

The *Cas9* gene was selected from *Streptococcus pyogenes* in this study, which was driven by the constitutive promoter of Pgpd, and transcriptionally regulated by the Tsdh.B terminator [9]. It has been reported that introducing a specific intron into the upstream of the *Cas9* gene further enhanced its expression efficiency [11]. Moreover, to improve the nuclear localization capability of the Cas9 protein, NLS sequences from SV40 (simian vacuolating virus 40) were incorporated at both ends of the *Cas9* gene in a previous study [13]. In the same study, the U6 promoter was chosen to drive sgRNA expression, and an HDV ribozyme element was added to the sgRNA terminus, followed by linkage of the SUP4 terminator to ensure efficient processing and stability. Based on the above findings, we constructed a CRISPR/Cas9 gene editing vector by recombining seven cassettes as the gene-editing enhancement elements in an arrangement of Pu6-HDV-Tsup4-Pgpd-NLS-intron-Cas9 in the present study (Figure 1).

Our gene-editing vector mainly consists of four parts: the *Cas9* gene expression module, the sgRNA expression module, the hygromycin resistance gene (*hyg*) expression module, and the prokaryotic screening module, which are constructed as follows. The *Cas9* gene, designed on the basis of the *Streptococcus pyogenes* sequence after codon optimization, is composed of 4104 deoxyribonucleotides. To facilitate the sgRNA assembly, a pre-reserved Pu6-HDV-Tsup4 expression cassette sequence, in which a cutting site of the restriction enzyme Kpn1 was specifically introduced within the region between Pu6 and HDV for the following ligation of synthesized sgRNA1 and sgRNA2 fragments, was then integrated into the upstream region of the *Cas9* gene. After the synthesis of DNA sequences designed as above (Weimi Biotechnology Co., Ltd., Changzhou, China) using the vector pYL156 (GenBank: OP757700.1) as a backbone, an expression fragment carrying a synthesized sgRNA1-HDV-Tsup4-Pu6-sgRNA2 sequence was finally constructed and inserted into the *Cas9* expression vector between the Pu6 and HDV sequences, thereby to complete the assembly of the entire Cas9 gene-editing vector, which was named as P203535. The full sequence of the above Cas9 gene-editing vector was transformed into *Escherichia coli* DH5α cells for cloning and amplification, using kanamycin as a screening marker (Figure S1). Table 1 shows the sequences of two sgRNAs used for each gene editing that are selected using a method present at http://crispor.tefor.net/ (accessed on 6 March 2024).

### 2.5. PEG-Mediated Protoplast Transformation

PEG-mediated transformation was performed according to the methods described by Li et al. [23] and Yu et al. [24]. *G. sichuanense* strains were cultured on a liquid MYG medium after activation on the solid MYG medium. Mycelia growing in shake flasks after seven days were harvested and washed with sterile water and a 0.6 mol/L mannitol solution. Then, 2% (*w*/*v*) of lywallzyme (Institute of Microbiology, Guangdong Academy of Sciences, Guangzhou, China) was added for the enzymatic digestion of mycelium cells at 28 °C for 3.5 h. Protoplasts collected by centrifugation at 1200× *g* for six min were resuspended in STC (0.55 mol sorbitol, 10 mmol CaCl_2_, 10 mmol Tris-HCl, pH 7.5) and diluted to a concentration of 10^7^ protoplasts per 100 μL. A mixture solution of 100 μL protoplast suspension in STC, 1 μg DNA of gene editing vector, and 50 μL PTC (600 g/L PEG 4000, 10 mmol Tris-HCl, pH 7.5, 50 mmol CaCl_2_) was incubated on ice for 10 min. After that, 1 mL of PTC was added, mixed gently, and incubated again at room temperature for 30 min. The mixture was finally spread evenly onto a solid MYGA (yeast malt extract regeneration) medium containing 10 g/L maltose, 5 g/L yeast extract, 5 g/L glucose, and 15 g/L agar with a lethal concentration of hygromycin that was dependent on individual strains and incubated at 25 °C for 20 days as the first screening.

### 2.6. Screening and Identification of Genetically Edited Strains

Resistant colonies after the above transformation were transferred onto the MYG medium containing hygromycin for secondary screening. The transformants were subjected to five consecutive subcultures in the absence of selection pressure to ensure the stability of hygromycin resistance. Resistant transformants revealing consistent growth were randomly selected, and their genomic DNA was extracted using a DNA extraction kit (Kangwei biotech, Taizhou, China). PCR amplification and sequencing by using the primers designed with Primer5 from flanking regions of the target were used to verify the result of gene editing (Table 2). Gene editing efficiency was calculated by analyzing at least 50 randomly selected transformants.

### 2.7. Phenotypic Analysis of ura3 Gene Deletion Mutants

The mutants with identified *ura3* gene deletion were inoculated on both a PDA medium (Qingdao Hope Bio-technology, Qingdao, China) and a PDA uracil supplement medium that contains 100 mg/L uracil adenosine (Sangon Biotech, Shanghai, China) and 200 mg/L uracil (Sangon Biotech, Shanghai, China). All cultures were carried out within an incubation room at 25 °C in darkness for five days to observe their difference on mycelium growth.

## 3. Results

### 3.1. Evaluation of Hygromycin B Lethal Concentration

**Figure 2:**
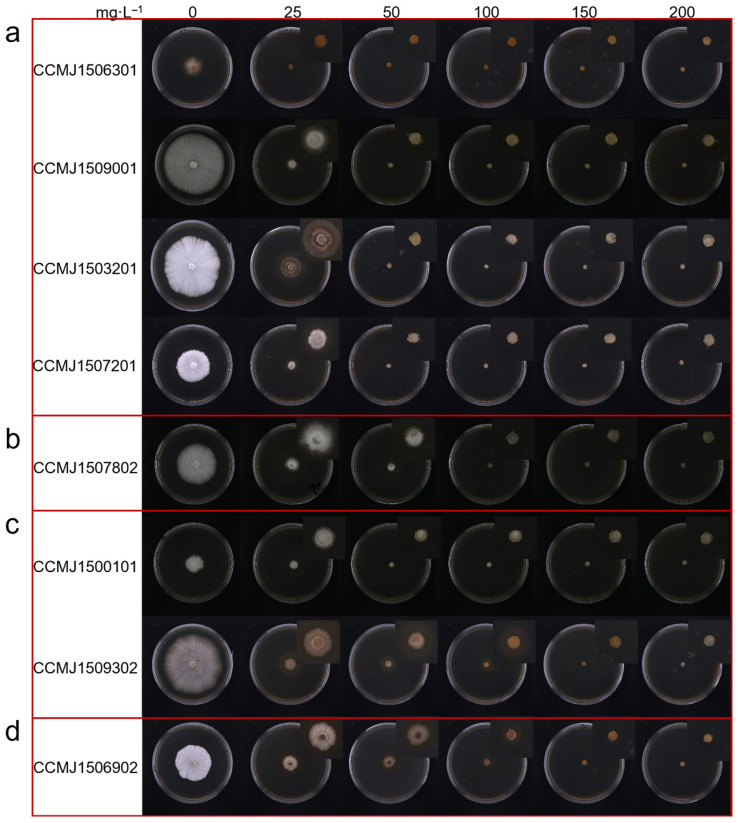
Evaluation of hygromycin B lethal concentration. (**a**) highly sensitive strains; (**b**) moderately sensitive strains; (**c**) moderate-tolerance strains; (**d**) strong-tolerance strains.

A hygromycin B inhibition assay was performed to determine the lethal concentration for eight *G. sichuanense* monokaryotic strains. The resulting inhibition concentrations ranged from 50 to 200 mg/L. (Figure 2): Strains CCMJ1506301, CCMJ1509001, CCMJ1503201, and CCMJ1507201 were completely inhibited at 50 mg/L, indicating highly sensitivity. Strain CCMJ1507802 was inhibited at 100 mg/L, indicating moderate sensitivity. Strains CCMJ1500101 and CCMJ1509302 were inhibited at 150 mg/L, indicating moderate tolerance. Strain CCMJ1506902 was not fully inhibited until the concentration reached 200 mg/L, indicating strong tolerance. Interestingly, monokaryotic strains isolated from the same dikaryons, such as CCMJ1503201 (sensitive) and CCMJ1500101 (moderately tolerant), CCMJ1506301 (highly sensitive), CCMJ1506902 (strongly tolerant), CCMJ1507201 (highly sensitive), and CCMJ1507802 (moderately sensitive), show a valid difference in hygromycin B sensitivity.

Using hygromycin B as the selective marker, only *Ganoderma* strains that are sensitive to moderately sensitive (completely inhibited at 50 to 100 mg/L) are suitable for genetic transformation. Our findings reveal the extensive genetic diversity of hygromycin B inhibition among *Ganoderma* strains, and thus provide critical guidance for selecting more suitable recipient strains in genetic transformation experiments.

### 3.2. Screening of Genetically Transformed Ganoderma Strains

**Figure 3:**
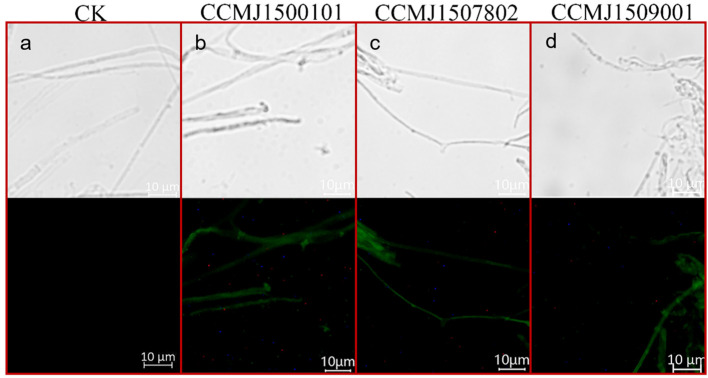
Results of fluorescent protein microscopic detection. (**a**) Optical and fluorescent microscopy images of Strain CCMJ1500101 without plasmid P203521; (**b**) Optical and fluorescent microscopy images of Strain CCMJ1500101 harboring plasmid P203521; (**c**) Optical and fluorescent microscopy images of Strain CCMJ1507802 harboring plasmid P203521; (**d**) Optical and fluorescent microscopy images of Strain CCMJ1509001 harboring plasmid P203521.

Two selective markers were used to screen the transformants: hygromycin B and green fluorescence. Not only does the green fluorescence serve as a visual selective marker, but the strength of the fluorescence is also an indicator of how well the *G. sichuanense* strain is able to express heterologous proteins, such as Cas9. Successfully transformed strains should show significantly increased tolerance to hygromycin B and a robust green fluorescence signal. The transformation efficiency of the eight tested *G. sichuanense* strains varies remarkably; three strains, CCMJ1507802, CCMJ1509001, and CCMJ1500101, yielded PCR-positive transformants that consistently exhibited both increased hygromycin B resistance and a robust green fluorescence signal (Figure 3). The positive frequencies were 20.0%, 62.5%, and 66.7% (Figure S2). For strain CCMJ1506301, 10 out of 21 PCR-positive transformants showed increased hygromycin B resistance, but none exhibited a green fluorescence signal, suggesting the presence of potential post-transcriptional regulatory abnormalities or protein folding defects. For strain CCMJ1509302, all transformants initially exhibited increased resistance to hygromycin B, but subsequently lost this ability. This suggests either false positivity or the presence of an endogenous resistance gene. Strains CCMJ1503201, CCMJ1507201, and CCMJ1506902 failed to produce any valid transformants. Based on these results, strains CCMJ1500101, CCMJ1507802, and CCMJ1509001, which exhibited a consistently high transformation rate, were chosen for the subsequent gene-editing experiments.

### 3.3. Construction of CRISPR/Cas9 Gene Editing System

The *ura3* gene is used as an editing target. The enzyme that *ura3* encodes is essential for uracil synthesis in *G. sichuanense*. Its disruption creates an uracil-auxotrophic mutant that requires uracil supplementation for growth. The URA3 enzyme also converts 5-fluoroorotic acid (5-FOA) into the lethal compound 5-fluorouracil. Therefore, on a medium with both 5-FOA and uracil, wild-type cells die from 5-fluorouracil poisoning, while *ura3* knockout cells survive by avoiding toxin production and utilizing exogenous uracil. We exploited this unique property of the URA3 enzyme by targeting the *ura3* gene to validate our gene-editing system. A CRISPR/Cas9 gene-editing system was constructed. The *Cas9* gene was codon-optimized based on the codon usage bias of *Ganoderma*. Strain CCMJ1500101, which has demonstrated high *gfp* expression efficiency, was selected as the recipient.

**Figure 4:**
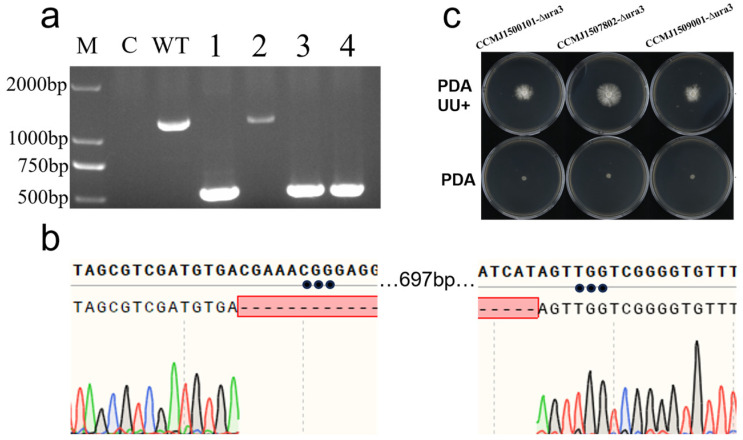
Results of *ura3* gene editing. (**a**) PCR verification of *ura3*-edited transformants. M: DNA marker DL2000; C: Blank Control (Water); WT: wild-type (WT); lanes 1–4: transformants; (**b**) sequencing chromatograms of PCR products from *ura3* transformants; (**c**) growth comparison of *ura3* transformants on uracil-deficient versus complete media.

The constructed Cas9-sgRNA expression vector was successfully introduced into host cells via PEG-mediated protoplast transformation at a density of 10^7^ cells/mL. Following hygromycin B selection, putative transformants were initially screened by PCR amplification of the full-length *ura3* gene. Gel electrophoresis revealed a clear size reduction in the amplicons from transformants (Figure 4a), which was further verified by sequencing to confirm a 697-bp deletion as expected within the targeted region (Figure 4b). Based on these results, the gene editing efficiency in strain CCMJ1500101 was calculated as 75.0% (Figure S3). Phenotypic assays showed that, compared to the wild-type strain (Figure S4), the mutants grew on PDA supplemented with uridine and adenine but failed to grow on minimal medium (Figure 4c), which validates the successful knockout of the *ura3* gene.

### 3.4. Applications of the CRISPR/Cas9 Gene Editing System in Other Ganoderma Strains

Using the same *ura3* target and PEG-mediated CRISPR/Cas9 system, parallel experiments were conducted in the wild strain CCMJ1509001 and the cultivated strain CCMJ1507802. The purpose of these parallel experiments is to test the universality of the editing system. PCR analysis verified successful *ura3* editing in both strains, with efficiencies of 54.6% and 14.3%, respectively. These results demonstrate that the established editing system is applicable across diverse genetic backgrounds, but its efficiency exhibits significant strain-dependent variation. Interestingly, gene inversion was observed for the first time in positive transformants: two from CCMJ1509001 and one from CCMJ1507802. This structural variation, not detected in the model strain CCMJ1500101, suggests the presence of specific DNA repair mechanisms in different genetic backgrounds.

### 3.5. Applications of the CRISPR/Cas9 Gene Editing System in Other Genes

**Figure 5:**
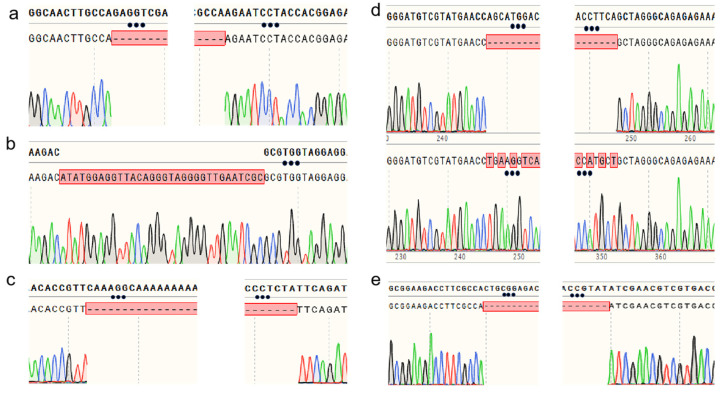
Sequencing verification of gene-edited transformants: (**a**) Sequence alignment of the *GsCYP5359E2* gene in transformants; (**b**) Comparative sequencing results of the *GsCYP5359C1* gene in transformants; (**c**) Sequencing results of the *GsCYP5359AA1* gene; (**d**) Sequence alignment of the *Gshd1* gene; (**e**) Sequence alignment of the *Gshd2* gene.

The CYP5359 family is unique to *Ganoderma* sp. [25]. To further verify the applicability of the CRISPR/Cas9 gene editing system in *G. sichuanense*, three cytochrome P450 genes (*GsCYP5359E2*, *GsCYP5359C1*, and *CYP5359AA1*) belonging to the CYP5359 family were chosen as the targets instead of *ura3*. Strain CCMJ1500101 was used as the recipient material. All essential experimental steps were conducted in the same procedures as those applied for the *ura3* gene. Table 1 shows the sgRNA sequences designed respectively for *GsCYP5359E2* and *GsCYP5359C1* genes of *G. sichuanense*. As a result of gene editing for *GsCYP5359E2*, a 201-bp deletion (from the 16th base pair upstream of the start codon to the 185th base pair on its downstream) was detected and verified by the sequencing (Figure 5a). For *GsCYP5359C1*, a 140-bp deletion (from the 28th to 168th base pair of the start codon) was expected; however, sequencing revealed a 32-bp segment insertion at the sgRNA2 site instead (Figure 5b). The upstream sequence of the *CYP5359AA1* gene (−433 bp to −176 bp relative to the start codon) in strain CCMJ1500101 was also targeted. PCR amplification and sequencing of the inter-sgRNA region revealed two distinct edit types: the expected 257-bp deletion and a sequence inversion (Figure 5c).

The mating-type A (mat A) locus homeodomain 1 (HD1) and homeodomain 2 (HD2) commit mated cells to sexual development [26]. Having established editing in metabolic genes, we extended the application of our CRISPR/Cas9 system to the mating-type regulatory genes *Gshd1* and *Gshd2* in strain CCMJ1500101, to further demonstrate its broad utility. For *Gshd1*, sequencing analysis revealed a 107-bp deletion downstream of the sgRNA1 (spanning nucleotides 60–167) in some of the transformants, leading to a frameshift mutation (Figure 5d), while others exhibited a local sequence inversion. For *Gshd2*, all transformants carried a uniform 160-bp frameshift deletion downstream of the sgRNA1 (Figure 5e). This consistent deletion pattern suggests the presence of a conserved editing site within the *Gshd2* gene region.

## 4. Discussion

Transformation efficiency may vary among different genotypes, even with the same species. In the plant kingdom, the genotype of a recipient has been considered as a crucial factor that might hardly be overcome or complemented through the optimization of external factors [27]. *G. sichuanense* is a fungus showing high genetic diversity with significant sequence and structural variations among the different strains. The genotype of the plant recipient is considered a crucial factor that can hardly be overcome or complemented through optimizing other external factors [27]. Additionally, the heterokaryotic nature of *Ganoderma* sp., where multiple nuclei coexist in the cytoplasm of a single cell, further complicates its gene editing. This differs from plants and animals, which typically contain a single diploid nucleus per cell. During the gene editing in *G. sichuanense*, the Cas9-gRNA complex may enter into only one of the nuclei in a target cell rather than acting on both nuclei simultaneously. Although CRISPR/Cas9 has recently been applied for the gene editing in *G. lucidum* [9], its use in fact has been limited so far only to three strains with different genetic backgrounds, CGMCC 5.616, GL3315, and Hunong No. 1 [9,11,13,15,16,17,20,21,22]. To address these challenges in this study, we initially selected a total of eight monokaryotic strains carrying the different genotypes as recipients to ensure a high target coverage of the nuclear genomes. With the use of the above strains, a comprehensive genetic transformation system was established that enabled us to broadly evaluate the ability of these strains to stably inherit or express the foreign genes. Our results clearly revealed the presence of notable differences in the stability and expression activity of foreign genes among the different monokaryotic strains. Notably, the wild strain of CCMJ1500101 exhibited the highest efficiency of foreign gene expression and successful co-expression of the *hyg* and *gfp* genes, making it an ideal material for the construction of a fungal genetic operation platform. During the experiments, however, transformants obtained from the strain CCMJ1506301 were found to have no expression of the *gfp* gene.

With the advancement and application of gene editing technology, studies have shown that incorporating specific components into editing vectors can remarkably enhance the gene editing efficiency [11]. By integrating multiple key elements to improve editing efficiency in this study, we successfully established a highly efficient and broad-spectrum dual-sgRNA in vivo transcription-based gene editing system. Building on the characteristics of endogenous introns in *G. sichuanense* was able to drive foreign gene expression [11]. We thus introduced an intron identified from the *G. sichuanense* gpd gene into the codon-optimized *Cas9* and employed the RNA polymerase III (Pol III) type U6 promoters to drive in vivo transcription of sgRNAs with a method as described before [11]. This system adopted a dual-sgRNA targeting strategy to combine with the HDV ribozyme processing of transcription products, and to incorporate NLS for enhancement of the nuclear import efficiency of the Cas9 protein. Successful knockout of the target gene *ura3* was achieved in three *G. sichuanense* strains, yielding 4, 11, and 7 positive transformants, respectively. The above results of this study are consistent with the previous reports in *G. lucidum*, in which use of T7 promoters to drive in vitro transcription of sgRNAs with different targeting sites of *ura3* resulted in three and five uracil auxotrophic strains [9], and the incorporation of HDV ribozyme processing led to an editing efficiency of 21.5% at the *ura3* locus [13]. For further enhancement of editing efficiency in *G. sichuanense*, we suggest that the codon usage frequency of *Cas9* should be matched with the translation machinery of *G. sichuanense*, and the copy number, type, and insertion position of NLS should be systematically optimized to ensure effective nuclear localization. It has been reported that this strategy could increase the editing efficiency by ninefold [28].

The advancement of the post-genomic era has greatly facilitated the analysis and functional validation of genes. In *Ganoderma* sp., traditional methods based on homologous recombination are often limited by low recombination efficiency. In contrast, newly developed techniques such as gene silencing and gene editing have been increasingly adopted for functional studies. In this study, we successfully established a gene editing system and achieved deletion of the *ura3* gene in three *G. sichuanense* strains, with editing efficiencies of 75.0%, 54.6%, and 14.3%, respectively. It is worth noticing that editing efficiency was notably higher in wild strains than in cultivated ones, suggesting the substantial influence of genetic background.

The CRISPR/Cas9 system has previously been used to characterize genes involved in terpenoid biosynthesis in *Gamoderma*, such as the ganoderic acid (GA) biosynthesis genes *cyp5150l8* and *cyp505d13* [13], the secondary metabolism regulator genes *glcrz1* and *glcrz2* [20], and the pyrimidine nucleotide biosynthesis gene *pyrG* [17]. The *Cyp505d13* gene, which encodes an enzyme involved in the bioproduction of squalene-type triterpenoid 2,3; 22,23-squalene dioxide, has also been disrupted [13]. In this study, we extend its utility by successfully editing three key GA-related genes: *GsCYP5359E2*, *GsCYP5359C1*, and *CYP5359AA1*.

The biosynthetic pathway of ganoderic acids in *G. lucidum* remains incompletely elucidated, particularly the roles of cytochrome P450 (CYP450) enzymes responsible for the oxidative modification of lanosterol, a key intermediate in GA biosynthesis [18]. For instance, *GlCYP5150L8* catalyzes the three-step oxidation of lanosterol to 3-hydroxy-lanosta-8,24-dien-26-oic acid (GA-HLDOA), *GlCYP5139G1* can further convert GA-HLDOA to 3,28-dihydroxy-lanosta-8,24-dien-26-oic acid (GA-DHLDOA), and *GlCYP512W2* catalyzes the conversion of GA-HLDOA to GA-Y and GA-Jb [29,30,31,32]. In the biosynthetic pathway of ganoderic acid, multiple genes from the CYP450 family have been revealed to be involved [33]. Consequently, gene-edited mutants of *GsCYP5359E2*, *GsCYP5359C1*, and *CYP5359AA1* will serve as excellent research materials for subsequent experiments. Therefore, the transformants obtained in this study should provide valuable materials for the subsequent functional validation of these genes in the terpenoid biosynthesis pathway. We also targeted two important mating-type genes, *Gshd1* and *Gshd2*. Both genes encode homeobox domain transcription factors. *G. sichuanense* is a tetrapolar species, meaning that the two distinct mating-type loci (mat A and mat B) control the development of dikaryotic hyphae formed after the fusion of two compatible monokaryons. Molecular analysis of the Agaricomycetes model species has demonstrated that the A mating type locus contains one or more divergently transcribed HD1–HD2 gene pairs [26]. The targeted editing of *Gshd1* and *Gshd2* in this study lays the groundwork for further understanding of the functional roles of mating-type genes in dikaryotization and fruiting body development in *G. sichuanense*. Use of a dual-sgRNA-mediated CRISPR/Cas9 system successfully led to the deletion of the *ura3* and *GL17624* genes in *G. lucidum* [11]. Gene editing using Cas9 and in vitro transcribed gRNAs, on the other hand, resulted in a gradual loss of antibiotic resistance during subculturing, indicating that the CRISPR-induced genomic instability and editing efficiency declined over time in that system [13].

In this study, gene disruption occurred not only through insertions and deletions but also via a newly observed phenomenon: inversion of the gene fragment without nucleotide insertions. In the editing of gene *GsCYP5359C1*, a 32-bp insertion bearing similarity to *Sus scrofa* sequences was detected, resembling the pattern previously reported in *G. lucidum*, where the disruption of the *pyrG* gene resulted from both deletions and insertions around the double-strand break (DSB) site [15]. Beyond insertions and deletions, our study also identified gene inversion events without nucleotide insertions when targeting the *Gshd1* gene in strain CCMJ1500101. One transformant in each group exhibited fragment inversion, strongly indicating that the excised fragment had been re-inserted in reverse orientation. Inversion was also observed during *ura3* editing in strains CCMJ1509001 and CCMJ1507802, where two and one transformants, respectively, carried inverted sequences. In basidiomycetes, double-strand DNA breaks are primarily repaired via the NHEJ pathway, which acts on specific cleavage sites [13]. Although NHEJ typically results in small insertions or deletions (indels), our findings suggest that it can also mediate fragment inversion during repair. These observations provide new insights into the NHEJ repair mechanism, particularly regarding naturally occurring inversions, which are known to play an important role in genome evolution [34]. Notably, various forms of chromosomal inversions have been reported in major crops such as rice [35], maize [36], and other species [37].

## 5. Conclusions

In conclusion, this study successfully established a CRISPR/Cas9 gene editing system in the important macrofungus *G. sichuanense*. Our results clearly demonstrate that gene-editing efficiency varies among different monokaryotic strains. Although further efforts are required to improve editing efficiency, the genome-edited mutants generated in this study provide valuable resources and essential materials for future research, which include functional gene characterization and investigations into the molecular and genetic mechanisms underlying growth, development, and terpenoid biosynthesis in *G. sichuanense*.

## Figures and Tables

**Table 1:** sgRNA sequences and plasmid for this study.

Gene Name	Plasmid Name	sgRNA1 (5′–3′)	sgRNA2 (5′–3′)
-	pYL156 (backbone)	-	-
*Gsura3*	P203535-*ura3*	5′-TTAGCGTCGATGTGACGAAAC-3′	5′-AATTGCGATGTCATCATAGT-3′
*GsCYP5359E2*	P203535-*CYP5359E2*	5′-GCTCTCCGTGGTAGGATTCT-3′	5′-AGGGAGTGGCAACTTGCCAG-3′
*GsCYP5359C1*	P203535-*Cyp5359C1*	5′-TTTGGCATCGGCGAGAATAT-3′	5′-CCCTTCCGTAGGAAGACGCG-3′
*GsCYP5359AA1*	P203535-*Cyp5359AA1*	5′-ACTTCCTTAACACCGTTCAA-3′	5′-TGGACCCGTCATCTGAATAG-3′
*Gshd1*	P203535-*hd1*	5′-TTTCTCTCTGCCCTAGCTGA-3′	5′-GGATGTCGTATGAACCAGCA-3′
*Gshd2*	P203535-*hd2*	5′-GCGGAAGACCTTCGCCACTG-3′	5′-GCGTCACGACGTTCGATATA-3′

**Table 2:** PCR primers for detecting gene editing events.

Name	Sequence (5′–3′)
testura3-F	ATGGTGGCCGTGGCCAAGC
testura3-R	CTAATCCGAGATCCCAACCC
testGsCYP5359E2-F	CTTCCGTCGTGACTGAGTGCTTC
testGsCYP5359E2-R	CGGAACGCTGGCTCCATGTCTGT
testGsCYP5359C1-F	GCGGATAGGAAGCCCTTTAGACT
testGsCYP5359C1-R	GACGCAGGACACGCAGCAGTATA
testGsCYP5359AA1-F	GAAACCACAGTCTCTCGCCAG
testGsCYP5359AA1-R	GCTAACTGTTTCATGCGTAGC
testGshd1-F	CGAGAGAACAACGGAGGATG
testGshd1-R	GAACCGGAGGCGAGACGAGAG
testGshd2-F	CTAAACAGACGGGCCGAAGC
testGshd2-R	TAATGAGCTTCTCATAGGGC

## Data Availability

The original contributions presented in this study are included in the article and Supplementary Materials. Further inquiries can be directed to the corresponding authors.

## Supplementary Materials

The following supporting information can be downloaded at: https://www.mdpi.com/article/10.3390/jof12010025/s1, Figure S1: Schematic Diagram of the URA3 Gene-Editing Vector Structure; Figure S2: PCR Verification of Partial Transformants Expressing the *gfp* Gene; Figure S3: PCR Verification of Partial URA3 Gene-Editing Transformants; Figure S4: Growth Status of Wild-Type GsURA3 Strain on PDA Medium and PDA Medium Supplemented with Uracil.
