# Supplementary material for: Screening of Monokaryotic Strains of Ganoderma sichuanense for Gene Editing Using CRISPR/Cas9
Source: J Fungi (Basel). 2025 Dec 28;12(1):25. doi: 10.3390/jof12010025 (PMC12842640; doi:10.3390/jof12010025)
Supplement: Supplementary file 1 [file jof-12-00025-s001.zip › jof-3976692-supplementary.pdf]

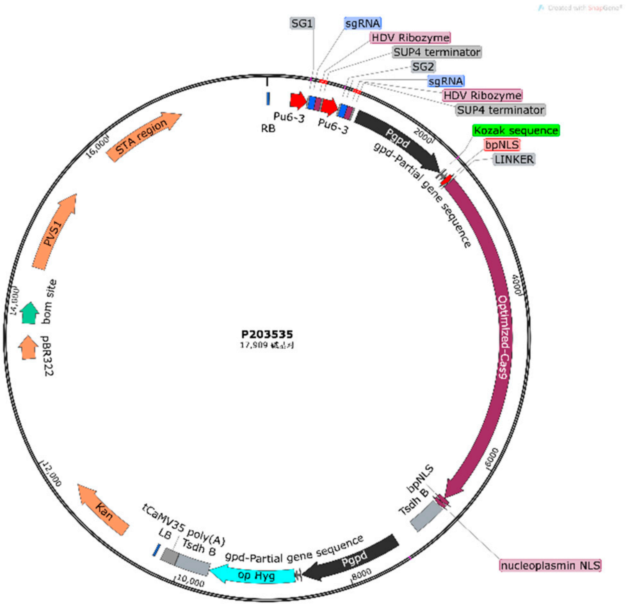

Figure S1 Schematic Diagram of the URA3 Gene-Editing Vector Structure

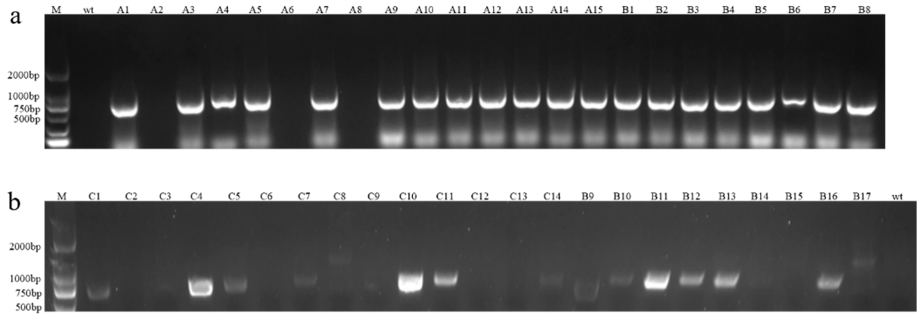

Figure S2 PCR Verification of Partial Transformants Expressing the *gfp* Gene. a: Detection Results for Strain CCMJ1500101 and CCMJ1509001 (M: DL2000 DNA; wt: Wild Type; A1-A15: Transformants of Strain CCMJ1500101; B1-B8: Transformants of Strain CCMJ1509001); b: Detection Results for Strain CCMJ1507802 and CCMJ1509001(M: DL2000 DNA; C1-C14: Transformants of Strain CCMJ1507802; B9-B17: Transformants of Strain CCMJ1509001; wt: Wild Type).

The wild-type strain yielded no band, whereas the positive transformants exhibited a distinct band.

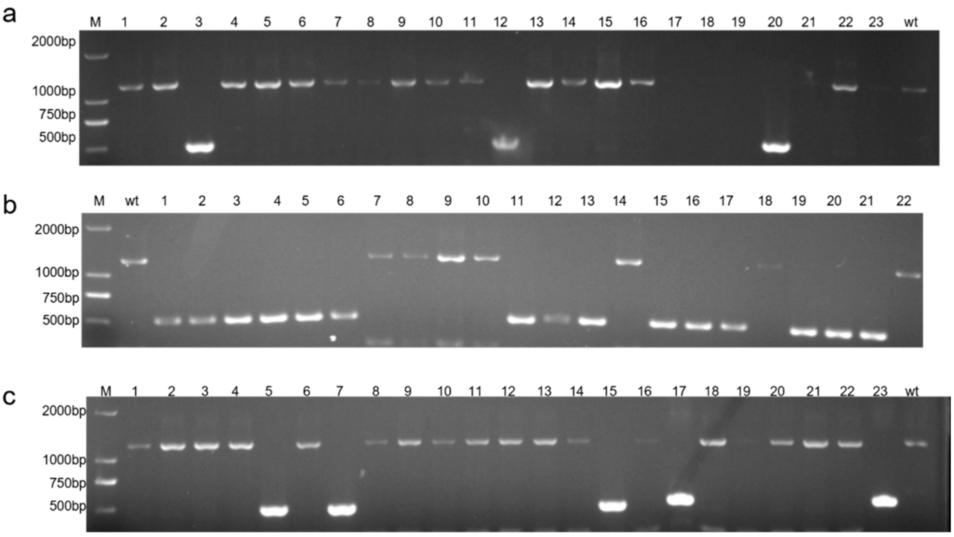

Figure S3. PCR Verification of Partial URA3 Gene-Editing Transformants，a. PCR Verification Results of Partial Transformants from Strain CCMJ1507802（M: DL2000 DNA ; 1-23: Transformants; wt: Wild Type）;b. PCR Verification Results of Partial Transformants from Strain CCMJ1500101（M: DL2000 DNA Marker1-22: Transformants; wt: Wild Type）; PCR Verification Results of Partial Transformants from Strain CCMJ1509001（M: DL2000 DNA Marker1-23: Transformants; wt: Wild Type）

A 679-bp smaller band was observed upon successful gene editing.

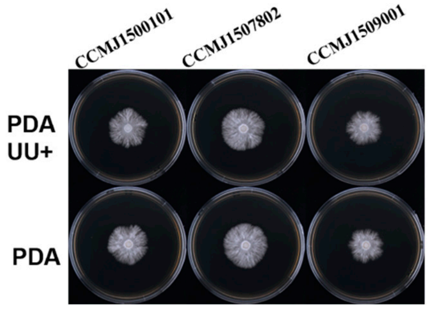

Figure S4 Growth Status of Wild-Type GsURA3 Strain on PDA Medium and PDA Medium Supplemented with Uracil

Sequencing Results of the Transformants:

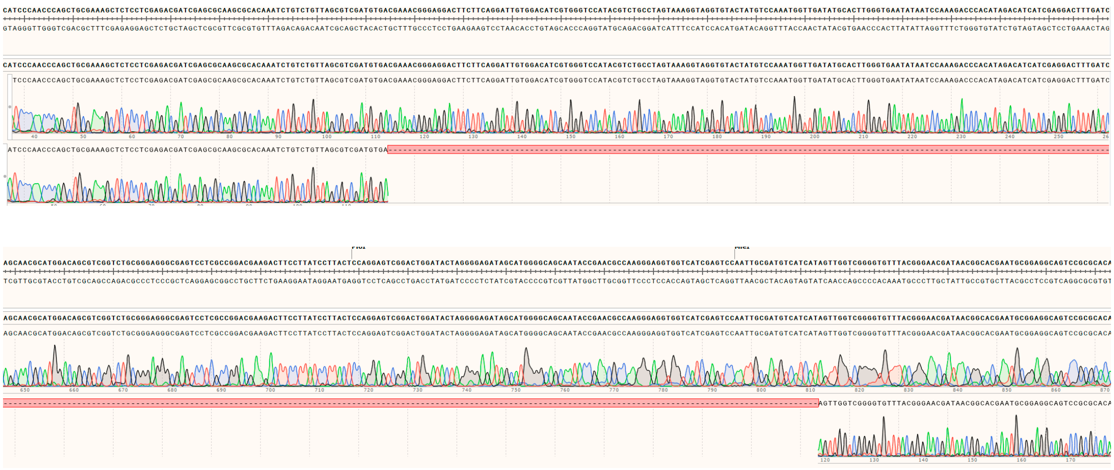

a . Sequencing Alignment Results of *URA3* Transformants

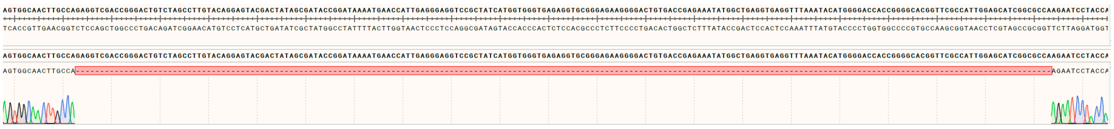

b. Sequencing Alignment Results of *CYP5359E2* Transformants

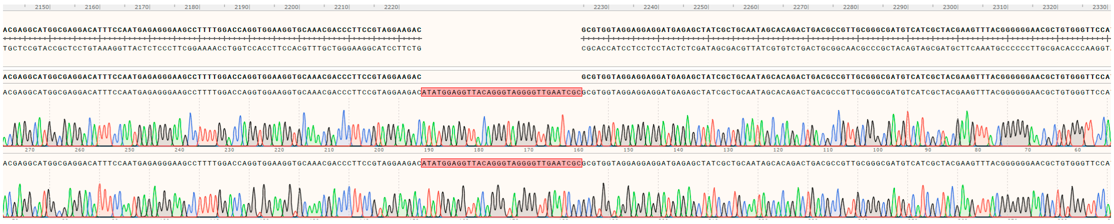

c. Sequencing Alignment Results of *CYP5359C1* Transformants

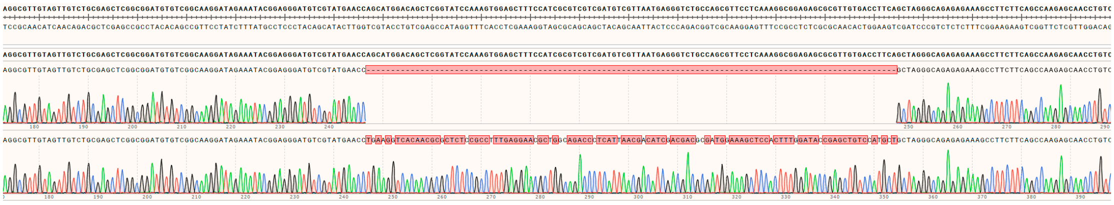

d. Sequencing Alignment Results of *GShd1* Transformants

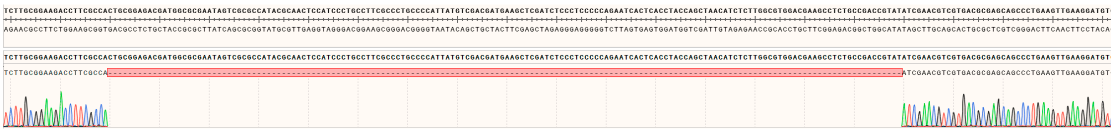

c. Sequencing Alignment Results of *Gshd2* Transformants
